# COVID-19 financial support to small businesses in Switzerland: evaluation and outlook

**DOI:** 10.1186/s41937-020-00060-y

**Published:** 2020-10-14

**Authors:** Marius Brülhart, Rafael Lalive, Tobias Lehmann, Michael Siegenthaler

**Affiliations:** 1 Department of Economics, HEC Lausanne, University of Lausanne, Lausanne, Switzerland; 2 Labour Market Section, KOF Swiss Economic Institute, ETH Zurich, Zurich, Switzerland

## Abstract

We analyse small businesses’ recourse to public support measures during the COVID-19 crisis using a survey of 1011 self-employed workers and small business owners in Switzerland. We find that “objective” measures of lockdown affectedness and economic structure explain fairly well how businesses availed of support measures to cover labour costs. Recourse to government-backed corona loans, however, appears to be driven to a larger extent by behavioural idiosyncrasies across firms. Specifically, previously indebted businesses took out corona loans more readily than those who had been debt-free before the pandemic. Since uptake is not well in line with firm fundamentals, we propose making loan repayments contingent on future profits. This will more effectively target and sustain businesses that are in trouble today but would be viable in the absence COVID-19.

## Introduction

Faced with rapidly increasing Covid-19 infections, the Swiss government decreed a partial lockdown on 16 March, 2020. Non-essential shops, restaurants, entertainment venues, schools, nurseries and universities were closed, and non-essential workers were asked to stay at home and work from there if possible. The lockdown was gradually relaxed after 27 April, with most restrictions lifted by 8 June. For many businesses, the pandemic meant a dramatic or even complete loss of turnover. Recent research shows that overall spending would have been severely reduced even in the absence of a decreed lockdown, because of consumers’ fear of infection and because of a collapse in export demand^1^. It is estimated that total demand fell by more than 30% during the Swiss lockdown, and GDP is projected to contract by more than 5% over the whole year^2^. Demand will likely remain subdued at least in certain sectors for as long as social distancing is warranted and foreign markets are depressed. This state of affairs could potentially take several years until a vaccine or an effective cure is found.

The combination of a time-limited forced shutdown of large parts of the economy followed by a potentially long lasting drag on contact-intensive activities presents economic policy-makers with monumental challenges. The main goals in the initial lockdown period were to avoid excessive income losses by workers in affected sectors and to avoid damage to the productive structure of the economy through large-scale layoffs and bankruptcies. Switzerland has been widely credited for meeting these aims successfully, through three essential policy tools: wage compensation for furloughed employees (short-term work, “Kurzarbeit”), income support for independent workers and small-business owners, and state-backed loans to cover businesses’ liquidity needs (corona loans). Rapid and un-bureaucratic intervention was possible thanks to Switzerland’s exceptionally healthy public finances, the prior existence of some of the relevant policy tools, a functioning banking sector, and a consensual, inclusive system of government.

In this paper, we shed light on the effectiveness of these tools in the early phase of the lockdown. To this end, we analyse data from a survey of 1011 independent workers and small- and medium-size firms conducted in mid-April 2020.

From this experience, we derive some implications for how economic policy measures could be adapted if the virus were to continue to constrain contact-intensive economic activities or even necessitate further lockdowns.

## Data and empirical model

Our empirical analysis builds on a survey conducted with LINK Institute, an established polling organisation in Switzerland. LINK have a nationwide, representative online participant sample of some 115,000 individuals, from which subsamples can be drawn for specific surveys. One attractive feature of this sampling environment is that a lot of baseline information is known about the participant population, which can then be correlated with the sample of individuals answering a specific survey^3^.

The survey sample underlying this paper was drawn from three sub-populations, as self-reported by the participants: the self-employed, the liberal professions and firm owners. The common denominator across these three sub-populations is that they can be considered as “job creators”—be it only for themselves or also for others. We shall refer to this population as “small businesses” or “businesses” in the remainder of this paper.

The survey was conducted between 14 and 20 April 2020. We had a response rate of 36%, which is satisfactory in this context. An analysis of non-response shows, perhaps surprisingly, no statistically significant differences in response rates across economic sectors and only minor differences in terms of individual characteristics. We find some signs of lower non-response rates by older individuals and by people with higher educational qualifications, but overall we are confident of the representativeness of our data sample (see also Brülhart, Klaeui, Lalive, Lehmann, & Siegenthaler, 2020b).

In total, we have answers from 1011 small businesses. Table 1 shows summary statistics. Sixty-three percent of respondents are based in German-speaking Switzerland, 26% in French-speaking Switzerland and 11% in Italian-speaking Switzerland. Fifty-nine percent of respondents are male, and the average age is 57. Fully 31% of surveyed businesses had availed of public subsidies for short-term work of employees, and 33% had availed of income replacement for independent workers and small business owners. Eighteen percent of sample businesses had availed or intended to avail of corona loans^4^.

**Table 1:** Summary statistics

Variables	Obs	Mean	Std. Dev.	Min.	Max.
*Dependent variables*					
Short-time work: used	1011	0.389	0.488	0	1
Short-time work: important	890	0.306	0.461	0	1
Income replacement: used	1011	0.411	0.492	0	1
Income replacement: important	892	0.333	0.472	0	1
Corona loans: used	1011	0.291	0.454	0	1
Corona loans: important	875	0.181	0.385	0	1
*Lockdown variables*					
Closed de jure	1011	0.405	0.491	0	1
Closed de facto	1011	0.256	0.437	0	1
Expected share of April turnover lost	935	− 0.436	0.459	− 1	1
Physical closeness important: workers	1011	0.428	0.495	0	1
Physical closeness important: clients	1011	0.641	0.480	0	1
*Economic variables*					
Employment					
1 FTE	1011	0.456	0.498	0	1
2–9 FTE	1011	0.396	0.489	0	1
> 10 FTE	1011	0.148	0.356	0	1
Debt ratio 2019					
0	904	0.676	0.468	0	1
0.01–0.25	904	0.140	0.348	0	1
0.26–1	904	0.079	0.270	0	1
Profit ratio 2019					
< 0	987	0.023	0.149	0	1
0–0.25	987	0.721	0.449	0	1
> 0.25	987	0.232	0.423	0	1
Owner private wealth 2019					
< 50k CHF	832	0.246	0.431	0	1
50k–500k	832	0.388	0.487	0	1
> 500k CHF	832	0.189	0.392	0	1
Labour cost share 2019					
< 0.33	935	0.465	0.499	0	1
0.33–0.66	935	0.257	0.437	0	1
0.66–1	935	0.203	0.402	0	1
*Behavioural variables*					
German-speaking Switzerland	1011	0.630	0.483	0	1
French-speaking Switzerland	1011	0.261	0.439	0	1
Italian-speaking Switzerland	1011	0.109	0.312	0	1
Age					
< 45	989	0.138	0.345	0	1
45 ≤ age ≤ 65	989	0.694	0.461	0	1
> 65	989	0.169	0.375	0	1
Education					
Compulsory school only	1006	0.017	0.129	0	1
Vocational qualifications	1006	0.322	0.468	0	1
Academic qualifications	1006	0.656	0.475	0	1
Female	1011	0.411	0.492	0	1
Swiss national	1011	0.812	0.391	0	1

The aim of our data analysis is to explore the determinants of small businesses’ recourse to those different sources of financial support. We consider three sets of explanatory factors. All but one of the regressors are converted into binary variables for ease of interpretation:

1. *Lockdown variables*: These variables are included to capture the impact of the pandemic on the activity of the sample businesses. They should therefore correlate strongly with those businesses’ use of support measures. We consider the following lockdown variables:
  1.1 Whether a business was locked down *de jure* (i.e. by government decree),
  1.2 Whether a business was locked down *de facto* (i.e. in principle allowed to operate and open, but severely restricted in its activity because of a lack of custom and/or because of sanitary regulations),
  1.3 The share of April 2020 turnover that respondents estimate to have lost due to the pandemic (the only non-binary variable),
  1.4 Whether physical proximity amongst workers is important for the activity of the business, and
  1.5 Whether physical proximity to customers is important for the activity of the business.
2. *Economic variables*: These variables are included to capture pre-crisis characteristics of the sample businesses that can be expected to affect the extent to which they avail of different support measures. They are mainly designed to capture the financial capacity of small businesses and are therefore also expected to correlate with businesses’ use of support measures. We consider the following economic variables:
  2.1 Employment (1, between 2 and 9, or more than 10 full-time equivalent jobs, (FTE)),
  2.2 Debt ratio in 2019 (0, between 1% and 25% or more than 25% of own capital),
    Profit ratio in 2019 (less than 0, between 0 and 25%, or more than 25% of total revenue),
  2.3 Owner’s private net wealth in 2019 (less than CHF 50,000, between CHF 50,000 and CHF 500,000, more than CHF 500,000), and
  2.4 Share of labour costs in total costs in 2019 (less than 33%, between 33% and 66%, more than 66%).
3. *Behavioural variables*: These variables are included to capture characteristics of the sample businesses and their owners that cannot, on standard economic grounds, be expected to affect the extent to which those businesses avail of different support measures. They are therefore not expected to correlate strongly with businesses’ use of support measures, conditional on controlling for the lockdown and economic variables. We consider the following behavioural variables:
  3.1 Linguistic region (German, French or Italian-speaking Switzerland),
  3.2 Age of the respondent (below 45, between 45 and 65, over 65),
  3.3 Educational attainment of the respondent (compulsory school only, vocational qualifications, academic qualifications),
  3.4 Gender of the respondent (1 = female), and
  3.5 Nationality of the respondent (1 = Swiss).

In addition, we also consider the inclusion of fixed effects for 13 broad sectors^5^.

Using these variables, we estimate variants of the following multiple regression model:

1 $$ {Y}_{ij}^{\mathrm{measure}}={\beta}_0+{\boldsymbol{\upbeta}}_{\mathrm{ldown}}^{\mathrm{measure}}{\mathbf{X}}_{ij}^{\mathrm{ldown}}+{\boldsymbol{\upbeta}}_{\mathrm{econ}}^{\mathrm{measure}}{\mathbf{X}}_{ij}^{\mathrm{econ}}+{\boldsymbol{\upbeta}}_{\mathrm{behav}}^{\mathrm{measure}}{\mathbf{X}}_{ij}^{\mathrm{behav}}+{\gamma}_j+{\epsilon}_{ij}^{\mathrm{measure}}, $$

where ${Y}_{ij}^{\mathrm{measure}}$ is a binary variable capturing whether small business *i* in sector *j* has availed of the relevant policy measure, the three **X**_*ij*_ vectors contain, respectively, lockdown, economic and behavioural independent variables with their associated coefficient vectors **β** estimated with OLS, *γ*_*j*_ is a sector fixed effect, and *ϵ*_*ij*_ is a random error.

## Results and discussion

### Results

**Fig. 1:**
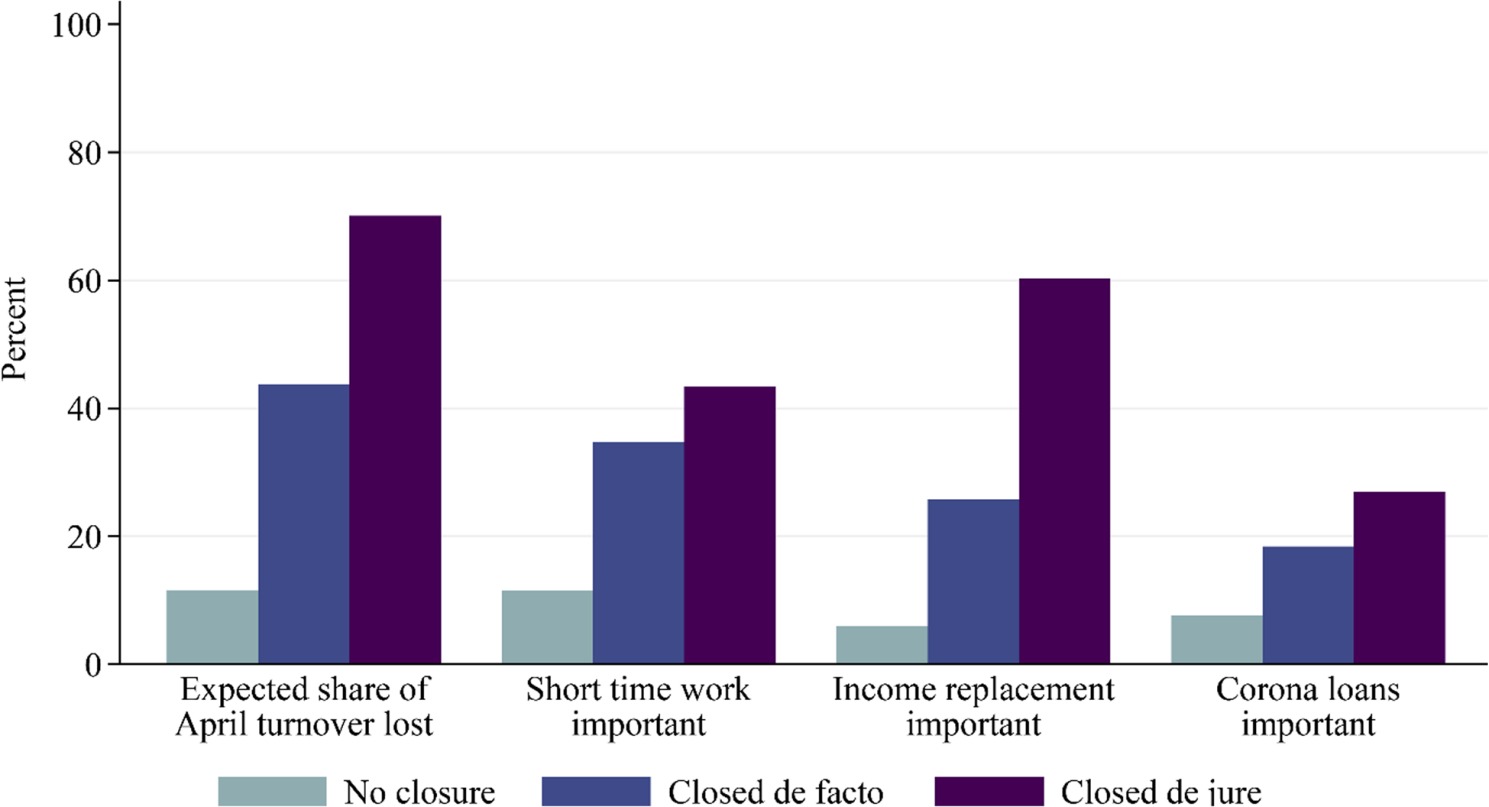
Reported loss of turnover and recourse to support measures

Figure 1 shows that businesses closed by government decree (*de jure*) on average expected to lose 70% of their revenue in April, whereas businesses that could remain open without major restrictions reported revenue losses of 10% on average. Closed businesses also availed much more of all three policy measures, “short-time work”, “income replacement” and “corona loans”. The contrast is largest for the “income replacement” measure, which was used by 60% of businesses closed *de jure* and by only 6% of businesses that could remain open without major restrictions.

These unconditional statistics show that the take-up of government support measures was clearly driven by firms’ lockdown affectedness. But affectedness explains only part of the take-up: not all closed businesses availed of the measures, and many businesses that remained in operation did. We therefore turn to multiple regression analysis in order to explore alternative determinants of firms’ recourse to public support.

**Fig. 2:**
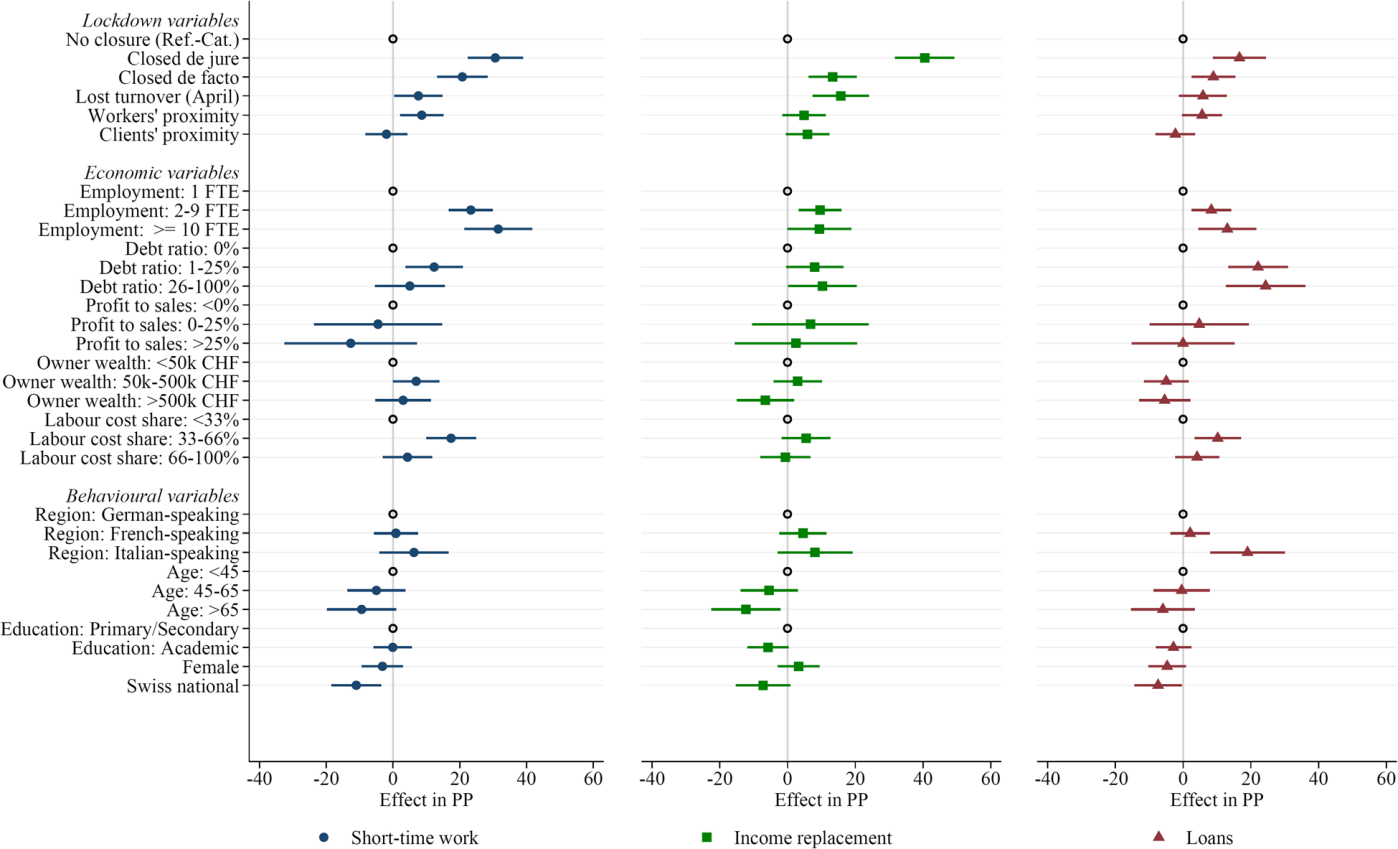
Determinants of the use of support measures (illustration of estimated coefficients). The graphs show coefficient estimates and 95% confidence intervals as reported in column (5) of Tables 2, 3 and 4

In Tables 2, 3 and 4, we report our estimates of different variants of Eq. (1). We consider three dependent variables, one for each of the policy measures we study: “short-time work” (Table 2), “income replacement” (Table 3) and “corona loans” (Table 4). A graphical illustration of our main coefficient estimates is provided in Fig. 2.

**Table 2:** Determinants of the use of the short-term work

*Independent variables:*	(1)	(2)	(3)	(4)	(5)	(6)
Closed *de jure*	0.27***	0.25***	0.32***	0.29***	0.31***	0.28***
	(0.05)	(0.05)	(0.04)	(0.04)	(0.04)	(0.04)
Closed* de facto*	0.19***	0.18***	0.21***	0.19***	0.21***	0.18***
	(0.04)	(0.04)	(0.04)	(0.04)	(0.04)	(0.04)
Expected share of April turnover lost	0.06	0.06	0.07*	0.07*	0.08**	0.07**
	(0.04)	(0.04)	(0.04)	(0.04)	(0.04)	(0.04)
Physical closeness important: workers	0.19***	0.18***	0.09***	0.10***	0.09***	0.10***
	(0.03)	(0.03)	(0.03)	(0.03)	(0.03)	(0.03)
Physical closeness important: clients	− 0.03	− 0.02	− 0.02	− 0.04	− 0.02	− 0.04
	(0.04)	(0.04)	(0.03)	(0.03)	(0.03)	(0.03)
Employment: 2–9 FTE			0.23***	0.22***	0.23***	0.23***
			(0.03)	(0.03)	(0.03)	(0.03)
Employment: > 10 FTE			0.32***	0.31***	0.32***	0.31***
			(0.05)	(0.05)	(0.05)	(0.05)
Debt ratio 2019: [0.01–0.25]			0.12***	0.12***	0.12***	0.13***
			(0.04)	(0.04)	(0.04)	(0.04)
Debt ratio 2019: [0.26–1]			0.06	0.09	0.05	0.08
			(0.05)	(0.06)	(0.05)	(0.06)
Profit to sales ratio 2019: [0–0.25]			− 0.05	− 0.04	− 0.04	− 0.03
			(0.10)	(0.10)	(0.10)	(0.10)
Profit to sales ratio 2019: > 0.25			− 0.13	− 0.12	− 0.12	− 0.11
			(0.10)	(0.11)	(0.10)	(0.10)
Owner private wealth 2019: [50k–500k CHF]			0.06	0.07*	0.07*	0.08**
			(0.04)	(0.04)	(0.04)	(0.04)
Owner private wealth 2019: > 500k CHF			0.01	0.00	0.03	0.03
			(0.04)	(0.04)	(0.04)	(0.04)
Labour cost share 2019: < 0.33			− 0.04	− 0.04	− 0.04	− 0.04
			(0.04)	(0.04)	(0.04)	(0.04)
Labour cost share 2019: [0.33–0.66]			0.14***	0.13***	0.13***	0.13***
			(0.04)	(0.04)	(0.04)	(0.04)
German-speaking Switzerland					0.01	− 0.00
					(0.03)	(0.03)
Italian-speaking Switzerland					0.06	0.04
					(0.05)	(0.05)
45 ≤ age ≤ 65					− 0.05	− 0.06
					(0.04)	(0.04)
Age > 65					− 0.10*	− 0.11**
					(0.05)	(0.05)
Education: vocational qualifications					0.08	0.04
					(0.12)	(0.12)
Education: academic qualifications					0.07	0.03
					(0.12)	(0.12)
Female					− 0.03	− 0.02
					(0.03)	(0.03)
Swiss national					− 0.11***	− 0.10***
					(0.04)	(0.04)
Observations	822	822	822	822	822	822
Adjusted *R*^2^	0.12	0.16	0.28	0.29	0.28	0.30
Industry FE	No	Yes	No	Yes	No	Yes

All dependent and independent variables are binary (0/1), except for “Expected share of April turnover lost”. The dependent variable equals 1 for businesses that describe short-term work as “highly important” or “of intermediate importance”, and it equals 0 if or businesses that describe short-term work as “not used” (businesses describing short-term work as “weakly important” are omitted). Parameters are estimated by means of a linear probability model (OLS), with robust standard errors (in parentheses, ***p < 0.01, **p < 0.05, *p < 0.1)

**Table 3:** Determinants of the use of income replacement

*Independent variables:*	(1)	(2)	(3)	(4)	(5)	(6)
Closed *de jure*	0.41***	0.39***	0.42***	0.40***	0.41***	0.39***
	(0.04)	(0.05)	(0.04)	(0.05)	(0.04)	(0.05)
Closed *de facto*	0.11***	0.11***	0.13***	0.12***	0.13***	0.12***
	(0.04)	(0.04)	(0.04)	(0.04)	(0.04)	(0.04)
Expected share of April turnover lost	0.15***	0.14***	0.15***	0.14***	0.16***	0.15***
	(0.04)	(0.04)	(0.04)	(0.04)	(0.04)	(0.04)
Physical closeness important: workers	0.09***	0.08**	0.06*	0.06*	0.05	0.05
	(0.03)	(0.03)	(0.03)	(0.03)	(0.03)	(0.03)
Physical closeness important: clients	0.06*	0.06*	0.06*	0.05	0.06*	0.04
	(0.03)	(0.04)	(0.03)	(0.03)	(0.03)	(0.03)
Employment: 2–9 FTE			0.09***	0.10***	0.10***	0.11***
			(0.03)	(0.03)	(0.03)	(0.03)
Employment: > 10 FTE			0.10**	0.09*	0.10**	0.09*
			(0.05)	(0.05)	(0.05)	(0.05)
Debt ratio 2019: [0.01–0.25]			0.08*	0.09**	0.08*	0.10**
			(0.04)	(0.05)	(0.04)	(0.04)
Debt ratio 2019: [0.26–1]			0.10**	0.13**	0.10**	0.14**
			(0.05)	(0.06)	(0.05)	(0.05)
Profit to sales ratio 2019: [0–0.25]			0.06	0.06	0.07	0.07
			(0.08)	(0.08)	(0.09)	(0.08)
Profit to sales ratio 2019: > 0.25			0.01	0.01	0.03	0.03
			(0.09)	(0.09)	(0.09)	(0.09)
Owner private wealth 2019: [50k–500k CHF]			0.00	0.00	0.03	0.03
			(0.04)	(0.04)	(0.04)	(0.04)
Owner private wealth 2019: > 500k CHF			− 0.12***	− 0.11***	− 0.07	− 0.06
			(0.04)	(0.04)	(0.04)	(0.04)
Labour cost share 2019: < 0.33			0.03	0.03	0.01	0.01
			(0.04)	(0.04)	(0.04)	(0.04)
Labour cost share 2019: [0.33–0.66]			0.08*	0.08*	0.06	0.06
			(0.04)	(0.04)	(0.04)	(0.04)
German-speaking Switzerland					0.05	0.05
					(0.04)	(0.04)
Italian-speaking Switzerland					0.08	0.08
					(0.06)	(0.06)
45 ≤ age ≤ 65					− 0.06	− 0.06
					(0.04)	(0.04)
Age > 65					− 0.13**	− 0.14***
					(0.05)	(0.05)
Education: vocational qualifications					0.10	0.09
					(0.13)	(0.13)
Education: academic qualifications					0.04	0.03
					(0.13)	(0.13)
Female					0.03	0.03
					(0.03)	(0.03)
Swiss national					− 0.07*	− 0.07*
					(0.04)	(0.04)
Observations	823	823	823	823	823	823
Adjusted *R*^2^	0.28	0.28	0.31	0.31	0.32	0.32
Industry FE	No	Yes	No	Yes	No	Yes

All dependent and independent variables are binary (0/1), except for “Expected share of April turnover lost”. The dependent variable equals 1 for businesses that describe income replacement as “highly important” or “of intermediate importance”, and it equals 0 if or businesses that describe income replacement as “not used” (businesses describing income replacement as “weakly important” are omitted). Parameters are estimated by means of a linear probability model (OLS), with robust standard errors (in parentheses, ***p < 0.01, **p < 0.05, *p < 0.1)

**Table 4:** Determinants of the use of corona loans

*Independent variables:*	(1)	(2)	(3)	(4)	(5)	(6)
Closed *de jure*	0.15***	0.15***	0.18***	0.17***	0.17***	0.15***
	(0.04)	(0.04)	(0.04)	(0.04)	(0.04)	(0.04)
Closed *de facto*	0.07**	0.08**	0.09***	0.09**	0.09***	0.08**
	(0.04)	(0.04)	(0.03)	(0.03)	(0.03)	(0.03)
Expected share of April turnover lost	0.06	0.07**	0.05	0.05	0.06	0.05
	(0.04)	(0.03)	(0.03)	(0.03)	(0.04)	(0.03)
Physical closeness important: workers	0.13***	0.11***	0.07**	0.07**	0.06*	0.06**
	(0.03)	(0.03)	(0.03)	(0.03)	(0.03)	(0.03)
Physical closeness important: clients	− 0.02	0.01	− 0.02	− 0.00	− 0.02	− 0.01
	(0.03)	(0.03)	(0.03)	(0.03)	(0.03)	(0.03)
Employment: 2–9 FTE			0.09***	0.06**	0.08***	0.07**
			(0.03)	(0.03)	(0.03)	(0.03)
Employment: > 10 FTE			0.13***	0.12***	0.13***	0.12***
			(0.04)	(0.04)	(0.04)	(0.04)
Debt ratio 2019: [0.01–0.25]			0.22***	0.22***	0.22***	0.22***
			(0.05)	(0.05)	(0.05)	(0.05)
Debt ratio 2019: [0.26–1]			0.25***	0.26***	0.24***	0.27***
			(0.06)	(0.06)	(0.06)	(0.06)
Profit to sales ratio 2019: [0–0.25]			0.05	0.05	0.05	0.04
			(0.07)	(0.07)	(0.07)	(0.07)
Profit to sales ratio 2019: > 0.25			0.01	0.01	0.00	0.00
			(0.07)	(0.07)	(0.08)	(0.08)
Owner private wealth 2019: [50k–500k CHF]			− 0.07**	− 0.06*	− 0.05	− 0.04
			(0.03)	(0.03)	(0.03)	(0.03)
Owner private wealth 2019: > 500k CHF			− 0.08**	− 0.07*	− 0.05	− 0.04
			(0.04)	(0.04)	(0.04)	(0.04)
Labour cost share 2019: < 0.33			− 0.04	− 0.04	− 0.04	− 0.04
			(0.03)	(0.03)	(0.03)	(0.03)
Labour cost share 2019: [0.33–0.66]			0.07*	0.07*	0.06	0.06
			(0.04)	(0.04)	(0.04)	(0.04)
German-speaking Switzerland					0.02	0.03
					(0.03)	(0.03)
Italian-speaking Switzerland					0.19***	0.19***
					(0.06)	(0.06)
45 ≤ age ≤ 65					− 0.00	0.00
					(0.04)	(0.04)
Age > 65					− 0.06	− 0.06
					(0.05)	(0.05)
Education: vocational qualifications					0.03	− 0.01
					(0.12)	(0.11)
Education: academic qualifications					0.00	− 0.01
					(0.12)	(0.11)
Female					− 0.05*	− 0.01
					(0.03)	(0.03)
Swiss national					− 0.07**	− 0.06*
					(0.04)	(0.04)
Observations	806	806	806	806	806	806
Adjusted *R*^2^	0.07	0.12	0.19	0.22	0.21	0.24
Industry FE	No	Yes	No	Yes	No	Yes

All dependent and independent variables are binary (0/1), except for “Expected share of April turnover lost”. The dependent variable equals 1 for businesses that describe corona loans as “highly important” or “of intermediate importance”, and it equals 0 if or businesses that describe corona loans as “not used” (businesses describing corona loans as “weakly important” are omitted). Parameters are estimated by means of a linear probability model (OLS), with robust standard errors (in parentheses, ***p < 0.01, **p < 0.05, *p < 0.1)

The results of the regressions are shown in Tables 2, 3 and 4. For each policy measure, the dependent variable is constructed as follows: we code as 1 all businesses who answered with either “of intermediate importance” or “highly important”, and as 0 all businesses who answered with “not used” (thus dropping businesses who answered “weakly important”)^6^.

For each dependent variable, we show estimates of six specifications: only lockdown explanatory variables, lockdown + economic explanatory variables and lockdown + economic + behavioural explanatory variables, each time without and with inclusion of sector fixed effects (coefficients not shown).

Rather than discussing each table and each of the 18 estimated regression models individually, we present results by focusing on what we consider to be the most important findings.

***Result 1: Overall, lockdown and economic variables have considerably more explanatory power than behavioural variables***

If we focus on specifications with industry fixed effects, adjusted *R*^2^ values of regressions that include only lockdown and economic variables range between 0.22 (corona loans) and 0.31 (income replacement). These are satisfactory values for a linear probability model, especially given the unavoidable imprecisions of survey responses. Moreover, the variables that are expected to correlate strongly with recourse to policy measures do correlate strongly. This lends plausibility to our data. It is also interesting to note that considering sector-specific dummies adds relatively little explanatory variation. This suggests that our basic set of control variables account well for the deterministic part of survey responses with respect to the take-up of policy measures.

Adding the behavioural variables in some instances does not increase the adjusted *R*^2^. The largest observed increase in adjusted *R*^2^ is by 0.02 (corona loans). Overall, therefore, “objective” affectedness was a much stronger determinant of support uptake than “subjective” behavioural aspects.

***Result 2: Amongst the lockdown variables, physical closeness of workers has a strikingly large effect on small businesses’ recourse to public support***

Amongst the lockdown measures, *de jure* closure has the strongest effect in the majority of regressions, and *de facto* closure also has a strong effect throughout. This is as expected. More surprising, perhaps, is the finding that physical closeness amongst workers has a large and statistically significant effect throughout, whereas physical closeness to clients appears as a weaker determinant of firms’ recourse to policy measures^7^. The result that businesses were more likely to avail of short-time work, *ceteris paribus*, if closeness amongst workers is important also suggests that the short-time work and income replacement programmes may have contributed to containing the virus by reducing contacts in the workplace.

***Result 3: Larger and indebted businesses are more likely to claim public support***

Amongst the economic variables, firm size and indebtedness have the largest impact overall. Businesses with more than one full-time employee have a higher probability of recourse to public support, *ceteris paribus*, than one-person firms. This pattern is stronger for short-term work than for income replacement, which adds further plausibility to the data, as the main policy measure available to one-person firms is income replacement. Similarly, the significant effect we observe for labour cost shares on short-time work conforms with expectations, as this is the policy measure designed to cover labour costs of crisis-hit firms.

The significantly lower take-up of corona loans by independent workers compared to businesses with more than one worker might also be related to legal liability: owners of sole proprietorships are liable for debt repayment with their private assets, whereas owners of incorporated businesses are liable only with their business assets. Hence, the latter may be more ready, other things equal, to take on corona loans^8^.

The strong effects of indebtedness on the likelihood of claiming income replacement and/or corona loans could reflect three patterns. First, it could capture the fact that these measures benefit in particular financially fragile businesses—i.e. those who need the measures most. Second, it could be the manifestation of indebted businesses having lower informational or attitudinal thresholds for accessing public support and for incurring further debt. And third, this result could arise from indebted firms replacing exiting debts with cheaper corona loans. Given that our regressions control for a range of variables related to financial fragility in the corona crisis (the lockdown variables, profit ratios, owner wealth), the second and third interpretations appear particularly plausible.

Result 4: The take-up of corona loans is statistically less well explained with lockdown and economic variables than the take-up of measures that target labour incomes

The explanatory power of the lockdown and economic factors as measured by adjusted *R*^2^ values (not considering fixed effects) is 0.19 for corona loans, whereas for short-term work it is 0.28 and for income replacement it is 0.31. Hence, recourse to corona loans appears to be more random, and less driven by “rational” lockdown and economic factors than the other two policy measures^9^.

It is particularly surprising that firms with a low *labour cost share* seem if anything to have below-average take-up of corona loans. Although this estimate could be biased towards zero because of imprecise measurement, it is concerning that the measure that comes closest to capturing the main target of the policy—i.e. sustaining businesses for which non-wage fixed costs account for a large share of outlays—has so little explanatory power.

There are moreover indications that certain unexpected business characteristics determine the take-up of corona loans. The strongest effect of all variables, stronger even than that of *de jure* closure, is observed on the variable *debt ratio*. This is suggestive of a sort of “habit effect”: firms already used to taking on debt are less reluctant towards taking on additional debt. It might also reflect some firms replacing existing debt with cheaper corona loans.

We observe that firms in Italian-speaking Switzerland resort to corona loans more than those in the rest of the country (controlling for lockdown severity, which was somewhat greater in the Italian part). There are also indications that businesses headed by men and businesses headed by non-Swiss nationals are somewhat more likely, *ceteris paribus*, to resort to corona loans.

### Discussion

Our results suggest that the take-up of corona loans across small businesses was more diffuse than that of labour support measures. Some measurable behavioural factors (especially prior history of indebtedness) seem to have played a role, and there appears to be considerable randomness in the process. This has implications both for “life-or-death” extensive-margin decisions and for “growth-vs-stagnation” intensive-margin decisions by private businesses. At the extensive margin, some firms that would be viable post-crisis and should thus avail of the subsidised loans may refrain from doing so, whilst some other firms take on cheap debt without needing to. At the intensive margin, firms saddled with corona debt may be prevented during or after the crisis from expanding in the way they would have in the absence of the pandemic.

The extensive-margin risk mainly consists of businesses folding because they are unwilling to take on extra debt and/or to burden future revenue streams with legacy costs from the COVID crisis. This risk could be mitigated by making loan repayment terms contingent on future profits, akin to student loans that only have to be repaid once graduates earn a certain income (Bonardi, Brülhart, Danthine, Jondeau, & Rohner, 2020). This idea has been further developed by Danthine, Fahlenbrach, and Morellec (2020). They propose that corona loans should be optionally convertible into preferred but non-voting equity stakes (“Vorzugsaktien”). This would turn fixed debt-repayment into flexible dividend payments, and it would free debtor firms from the constraints of a finite repayment period. Moreover, the discounted cost to the public purse of such a policy could well be close to zero^10^.

At the intensive margin, the main economic risk is that corona debt will lead to suboptimally low private investment. One measure towards reducing that risk is to lift the condition that corona loans may only be used to finance current spending but not capital spending or R&D spending (Gersbach, Mikosch, & Sturm, 2020). Moreover, for particularly hard-hit and difficult-to-transform businesses (e.g. hotels in certain tourist resorts), debt forgiveness based on sector/region-specific parameters could be envisaged (Bonardi et al., 2020), as well as public co-payments towards agreements between property owners and commercial renters (Brülhart et al., 2020a).

## Summary and outlook

The COVID-19 crisis has hit small businesses hard. Two thirds of our 1011 sample firms had to fully or partially cease operations during the height of the lockdown in April—40% because they had to close by government decree and 26% because remaining open was no longer commercially viable.

Moreover, the crisis afflicted small businesses randomly, in the sense that lockdown affectedness is not causally related to any measure of prior economic performance. This can be seen in the data. If we regress our main lockdown variables (*closed de jure*, *closed de facto* and *expected share of April turnover lost*) on the economic variables, the adjusted *R*^2^ values range between 0.00 and 0.02. The impact of the corona shock was clearly orthogonal to the prior financial viability of individual businesses.

When a historically large, entirely exogenous negative shock randomly affects large swathes of the private sector, public intervention is necessary in order to prevent both individual hardship and a macroeconomic slump. The fact that entire sectors were hit through no fault of their own by an exceptionally rare event also implied that moral hazard problems usually afflicting public support to businesses are much less of an issue in the COVID-19 crisis (Bonardi et al., 2020).

After it had announced the lockdown, the Swiss government moved swiftly to compensate affected workers and, to a lesser extent, businesses. Of our sample firms, 42% availed of at least one of the three measures (short-time work and income replacement to cover labour income, and corona loans to fill remaining liquidity needs), and 8% availed of all three^11^.

Our analysis suggests that the labour-income support measures reached their intended targets better than the corona loans: variables capturing the direct effects of the lockdown and the economic circumstances of individual businesses have greater explanatory power in the determination of who resorted to short-time work and income replacement than of who took out a corona loan. Take-up of corona loans was driven to a larger extent by firms’ prior history of indebtedness as well as by unrelated demographic and geographic variables.

Our evidence is consistent with cultural and behavioural factors playing a larger role in the take-up of loans than for the other measures. The data do not allow us to identify specific mechanisms, but it seems plausible to assume that deep-held attitudes and inhibitions to taking on debt vary across business owners. Differences in businesses’ acceptance of higher debt levels could mean that some viable businesses will shrink or exit because of an intrinsic aversion to debt, whilst some other firms will take on those subsidised loans without material need.

Moreover, some firms might have availed of corona loans to refinance their debts on more advantageous terms. To the extent that this could help some of those firms to weather the COVID storm, that effect is desirable. But there are likely to be firms for whom this represents a windfall that does not affect their survival prospects.

Looking ahead, the case for public generosity towards affected firms is growing weaker the longer the crisis continues. For a time-limited, government-imposed lockdown spanning a few weeks or months, the consensus view is that economic policy should aim at “freezing” productive capacity by plugging most of the private-sector revenue shortfalls through the public purse (e.g. Alós-Ferrer et al., 2020; Bonardi et al., 2020). Our estimates suggest that the policy measures taken by the Swiss government were effective in that respect.

Freezing cannot continue indefinitely, however, especially not once the economy is released from lockdown and consumer spending is picking up again (Brown et al. 2020). The longer some businesses’ pandemic-related problems persist, the higher is the likelihood that closing or restructuring them would be less costly in societal terms than keeping them afloat with public subsidies. Moral hazard issues return to the fore when the question becomes whether labour and capital should be allocated to more productive uses rather than remaining partly or fully idle in a “frozen” business.

This in turn implies that the compensation rate of policy measures should gradually be ratcheted down. The replacement rate of labour income measures could be lowered from the 80% currently applied (up to a ceiling), e.g. by 5% every quarter after the expiry of the current guaranteed period. Similarly, the part of any new corona loans that enjoys a state guarantee could be gradually lowered, and/or the interest rate charged could be increased.

Another approach could be to modulate the maximum duration with which wage support is granted. Kopp and Siegenthaler (2020) show that, during the financial crisis, most Swiss firms tended to leave the short-time work scheme before they reached the maximum benefit duration. However, the few firms that drew short-time work subsidies until the legal maximum laid off a significant share of previously subsidised workers. In these cases, the subsidies only postponed but did not prevent dismissals. These findings imply that phasing out government support can reduce the risk of funding ‘zombie jobs’. Short-time work support could be withdrawn differentially by economic sector, depending on affectedness by the pandemic.

Such a policy approach could help preserve viable businesses without preventing economically desirable firm closures or reorganisations^12^.

The main challenge for economic policy will be to find the right balance between supporting labour incomes and long-term viable businesses without preventing meaningful adaptation of the economy to the changed circumstances. It will be a delicate balancing act.

## Data Availability

Our data are available from the authors.

## Footnotes

1 See e.g. Brzezinski *et al*. (2020).

2 See SECO (2020) and KOF (2020).

3 This is a methodological advantage compared to one-off surveys with retrospective questions, such as that used in a related study by Zoller-Rydzek and Keller (2020). Their survey covered 205 managers of Swiss firms in the second week of April 2020. Due to differences in data collection and selection of explanatory variables, our findings are difficult to compare.

4 Respondents had four options. The percentages shown here are shares of respondents who declared the respective policy measure to be of average or high importance, as opposed to being of low or no importance (our “important” definition of takeup, see Table 1).

5 These sectors are: construction, education, art and culture, hotels and tourism, restaurants and bars, health, agriculture and gardening, information technology, non-food retail, food retail, hairdressing and beauty, legal and financial services, advertising and graphic design, and others (including small manufacturing).

6 This is the version “[Dependent variable]: important” as of Table 1. We ran the same regressions on the version “[Dependent variable]: used”, where the response category “weakly important” was coded as 1 instead of being dropped. The results are very similar (see the working paper version of this paper, Brülhart *et al*., 2020c).

7 The two sectors in which the largest shares of sample businesses indicated *physical closeness of workers* to be important are hairdressing and beauty (67%) and agriculture and gardening (52%). The two sectors with the smallest shares of sample business declaring *physical closeness of workers* to be important are information technology (21%) and graphic design and advertising (32%). The corresponding sectors for *physical closeness to clients* are hairdressing and beauty (100%) and healthcare (93%) at the upper end, and agriculture and gardening (33%) and information technology (34%) at the lower end.

8 We thank Cédric Tille for this insight. We cannot test the proposition directly, as the legal form of our sample businesses is not recorded in the data.

9 This finding is consistent with recent research on U.S. data, suggesting that the “Paycheck Protection Program” did not channel support to the hardest hit firms and had little effect on employment at small businesses (Granja *et al*., 2020; Chetty *et al*., 2020). The main problems in the U.S. case seem to be uneven access to banking services by small businesses and inefficient targeting of the policy at the most affected sectors. Given the different institutional settings, these do not appear to be plausible concerns in the context of the Swiss small-business support policies.

10 There may also be a case for reviewing the harsh Swiss insolvency laws. According to the World Bank’s Resolving Insolvency index, Switzerland ranks 49^th^ in the world, well behind Germany (4^th^), Italy (21^st^), Austria (22^nd^) and France (26^th^). As shown e.g. by Favara *et al*. (2016), bankruptcy codes that favour debt enforcement have a constraining effect on investment and growth of financially distressed firms.

11 The percentages are based on the “important” variant of our takeup measures.

12 A staggered policy drawdown would be comparable to “benefit frontloading” in unemployment insurance systems, whereby the replacement ratio is lowered during the unemployment spell (Hopenhayn and Nicolini, 1997; Lindner and Reizer, 2020). In the unemployment insurance context, benefit frontloading can have adverse moral hazard effects (Kolsrud *et al*., 2018). This is not a concern in the case of Covid support measures, where initial affectedness was exogenously determined, which further strengthens the case for a gradual lowering over time.

## References

[CR1] Alós-Ferrer, Carlos,* et al*. (2020) Coronavirus: testing and freezing – a survival strategy for the Swiss economy. *Mimeo*: Department of Economics, University of Zurich.

[CR2] Bonardi, Jean-Philippe, Marius Brülhart, Jean-Pierre Danthine, Eric Jondeau and Dominic Rohner (2020) The economics of wage compensation and corona loans: why and how the state should bear most of the economic cost of the COVID lockdown. *VoxEU.org*.

[CR3] Brown, Martin, Matthias Fengler, Rafael Lalive, Robert Rohrkemper and Thomas Spycher (2020) Monitoring consumption in Switzerland.* Online Dashboard* monitoringconsumption.org.

[CR4] Brülhart, Marius, Monika Bütler, Luca Crivelli, David Dorn, Jan-Egbert Sturm and Beatrice Weder (2020a) Public matching payments for commercial rent abatements during the COVID crisis. *Policy Brief*, Swiss National Covid-19 Science Task Force.

[CR5] Brülhart, Marius, Jeremias Klaeui, Rafael Lalive, Tobias Lehmann and Michael Siegenthaler (2020b). Covid Survey: Die Schweizer Selbständigerwerbenden in der Covid19-Pandemie. *Mimeo*: University of Lausanne and ETH Zürich.

[CR6] Brülhart, Marius, Rafael Lalive, Tobias Lehmann and Michael Siegenthaler (2020c) COVID-19 financial support to small businesses in Switzerland: evaluation and outlook. *KOF Working Paper* 483#, ETH Zurich. 10.1186/s41937-020-00060-y PMC7556580 33078128

[CR7] Brzezinski, Adam, Valentin Kecht and David Van Dijcke (2020) The cost of staying open – voluntary social distancing and lockdowns in the US. *Economics Working Paper* #910, University of Oxford.

[CR8] Chetty, Raj, John N. Friedman, Nathaniel Hendren and Michael Stepner (2020) How did COVID-19 and stabilization policies affect spending and employment? *Mimeo*: Harvard University.

[CR9] Danthine, Jean-Pierre, Rüdiger Fahlenbrach and Erwan Morellec (2020) Comment soutenir financièrement les PMEs suisses? Une solution durable visant à augmenter la resilience de l’économie. *Mimeo*, E4S (UNIL, EPFL, IMD), Lausanne.

[CR10] Gersbach, Hans, Heiner Mikosch and Jan-Egbert Sturm (2020) Mit einer Anpassung des COVID-19-Kreditprogramms gegen die Investitionsschwäche. *Oekonomenstimme.org*.

[CR11] Granja, Joao, Christos Makridis, Constantine Yannelis and Eric Zwick (2020) Did the paycheck protection program hit the target? *NBER Working Paper* #27095. 10.1016/j.jfineco.2022.05.006 PMC9409445 36042874

[CR12] Hopenhayn, Hugo A. and Juan Pablo Nicolini (1997) Optimal unemployment insurance. *Journal of Political Economy*, 105(2): 412-438.

[CR13] KOF (2020) Konjukturprognose, Mai 2020. *Report*, KOF Swiss Economic Institute, ETH Zurich.

[CR14] Kolsrud, Jonas, Camille Landais, Peter Nilsson and Johannes Spinnewijn (2018) The optimal timing of unemployment benefits: theory and evidence from Sweden. *American Economic Review*, 108(4-5), 985–1033.

[CR15] Kopp, Daniel and Michael Siegenthaler (2020) Short-time work and unemployment in and after the great recession. *Mimeo*: KOF Swiss Economic Institute, ETH Zurich.

[CR16] Lindner, Attila and Balázs Reizer (2020) Frontloading the unemployment benefit: an empirical assessment. *American Economic Journal: Applied Economics*, 12(3): 140-174.

[CR17] SECO (2020) Konjunkturprognose der Expertengruppe des Bundes – Juni 2020. *Report*, Staatssekretariat für Wirtschaft, Bern.

[CR18] Zoller-Rydzek, Benedikt and Florian Keller (2020) COVID-19: Guaranteed Loans and Zombie Firms. *Mimeo*: ZHAW School of Management and Law, Winterthur. 10.1093/cesifo/ifaa014 PMC7799295 34191927

